# Current News and Opinion

**Published:** 1882-03

**Authors:** 


### CURRENT NEWS AND OPINION.

SIR ROBERT CHRISTISON, died in Edinburgh, January 27th.

CHICAGO doctors have discovered a form of keratitis, due to malaria.

DR. KENNETH REID, of New York, died on January 22, of acute pericarditis.

THE NEW MICHAEL REESE HOSPITAL, of Chicago, is described as a model institution.

THE NEW *Scientific American* offices are located at 291 Broadway, corner of Warren Street.

M. NACHET, the noted French microscope maker died at Paris, December 28th, in his 53rd year.

M. BRIERRE DE BOISMONT, a very distinguished French alienist, died in December, aged 84 years.

AN Anti-vivisection league has been formed in this country. The first meeting was held in this city last month.

DR. M. S. DEAN, of Chicago, formerly president of the American Dental Association, died on January 28th.

Dr. Samuel Whitall died in this city, February 18th, of capillary bronchitis, complicated with malarial fever.

Fifty thousand dollars will probably be the total amount of the collections in this city on hospital Saturday and Sunday.

Dr. F. A. Mahomed has been appointed assistant physician, and Mr. C. J. Symonds assistant surgeon, to Guy's Hospital.

Six children is the latest effort of a California woman. The climate of the Pacific coast seems to be favorable to fertility.

A citizens' Sanitary Association has been formed in Savannah, Ga. Few cities in this country have been so badly in need of such an organization.

Billroth stretches the sciatic nerve subcutaneously. The patient is placed flat on his back, the leg is extended and the thigh strongly flexed upon the trunk.

Dr. Magitot says that a peculiar osteo-periostitis of the alveolar border of the jaw is a constant early and pathognomonic sign of diabetes mellitus.

Mr. John Flint South, the translator of Chelius' Surgery, an encyclopedic work, and an authority on surgery a half century ago, died on January 8th, in the 85th year of his age.

Dr. Samuel Green, for many years "city physician," of Boston, was recently elected Mayor of that city, by a combination of Democrats and Republicans, over the regular party nominees.

During the year 1881, the number of immigrants arriving from European ports at New York was 499,638. The total number of births en-route was 457, and the number of deaths 212.

Dr. D. G. Brinton, of Philadelphia, widely known as the editor of that excellent journal *The Medical and Surgical Reporter*, is one of the editors of the new literary weekly, *Our Continent.*

The miccroccus of lupus is the latest addition to our thesaurus of germs. Prof. Max Schuller, of Greifswald is the successful finder. The bacilli or micrococci of cancer and sarcoma still await a discoverer.

M. Bouley, the distinguished French veterinarian, has been created a commander of the Legion of Honor. This act of the French government contrasts strongly with the insolent smartness of the second-rate lawyer who recently asked an expert witness on the stand, if he was not "a horse doctor."

